# Woody plant encroachment drives the decline of a grassland bird: The fate of golden-shouldered parrot (*Psephotellus chrysopterygius*) nests

**DOI:** 10.1371/journal.pone.0327543

**Published:** 2025-07-23

**Authors:** Gabriel M. Crowley, Susan Shephard, Stephen A. Murphy, Stephen T. Garnett

**Affiliations:** 1 Department of Geography, Environment and Population, University of Adelaide, Adelaide, South Australia, Australia; 2 Artemis Station via Laura, Yarraden, Queensland, Australia; 3 Conservation Partners, Malanda, Queensland, Australia; 4 Research Institute for the Environment and Livelihoods, Charles Darwin University, Darwin, Northern Territory, Australia; UFERSA: Universidade Federal Rural do Semi-Arido, BRAZIL

## Abstract

Grasslands are the world’s most threatened terrestrial biome, with consequences for grassland-dependent species. Many remnant grasslands are threatened by woody plant encroachment (hereafter “encroachment”). Several studies have found that encroachment eliminates grassland species through increased predation rates at the nest. The golden-shouldered parrot *Psephotellus chrysopterygius* is an Endangered species found on Cape York Peninsula, Australia. Even though this species mainly nests along grassy drainage depressions, a previous study concluded that encroachment improves its nest success. We tested this proposition by assessing the fate of 555 eggs laid in 108 nests. We tested the impact of stem density on the fate of eggs, chicks and adults, and on predation events using linear-by-linear association tests; and on nest success using logistic exposure regression. We then compared the contraction of the parrot’s nesting distribution over three decades with change in canopy foliage cover. We also examined whether nest location was influenced by stem density, and explored the processes driving encroachment across the parrot’s distribution, particularly fire frequency, which has been shown to influence encroachment on Cape York Peninsula. The parrots preferentially nested in areas of low woody vegetation density. In contrast to previous work, we found that encroachment increased the probability of predation, and reduced nest success and survival of nesting adults. Encroachment both drove a decline in fire frequency and was exacerbated by it. The parrots have abandoned areas where encroachment has been most advanced. This study provides an Australian example of the negative effects of encroachment on nesting success that have been demonstrated in many species from North American prairies and other grassland habitats. It supports the current management efforts to reverse encroachment in the parrot’s habitat. We conclude that, wherever woody plant encroachment is occurring, it should be considered as a potential threatening process and managed accordingly.

## Introduction

Grasslands are recognised as the world’s most threatened terrestrial biome [1]. Even where they remain relatively intact and undeveloped, many remnant grasslands have been degraded by woody plant encroachment (hereafter “encroachment”) [2]. This process (also known as shrub encroachment or woody thickening) is driven by combinations of overgrazing, disruption to fire regimes, agricultural abandonment, climate change, carbon-dioxide fertilization, and trophic cascades that reduce the abundance of browsers of woody plants [3,4]. As well as threatening the grasslands themselves, encroachment adversely affects many of the species that require open habitat. It has been shown to alter species composition, diversity and richness [5–7], and is estimated to have reduced the population size of 121 bird species in southern African grasslands and savannas [8]. It is also recognised as contributing to the decline of several threatened birds (including the Critically Endangered Archer’s lark *Heteromirafra archeri* [9] and Vulnerable tawny eagle *Aquila rapax* [8] in Africa; and Vulnerable Sprague’s pipit *Anthus spragueii* and Vulnerable chestnut–collared longspur *Calcarius ornatus* in North America [10]).

Birds that depend on open environments often have lower breeding success when they build their nests near woody plants [11–17], frequently as a result of increased predation pressure [11,12,15,17]. For example, in North American prairies, nest failure in thick-billed longspur *Rhynchophanes mccownii* increased with proximity to shrubs, largely as a result of predation by thirteen-lined ground squirrel *Ictidomys tridecemlineatus* [11]; and daily nest survival of grasshopper sparrow *Ammodramus savannarum* and Henslow’s sparrow *A. henslowii* decreased with the amount of woody vegetation close to the nest as a result of predation by small-medium sized mammals [18]. In Israeli Mediterranean shrubland, encroachment by Aleppo pine *Pinus halepensis* reduced nest success of Sardinian warbler *Curruca melanocephala*, largely through increased predation by Eurasian jays *Garrulus glandarius* [15]. In Germany, predation at grey partridge *Perdix perdix* nests increased with the area of woodland around the nest [17].

The golden-shouldered parrot *Psephotellus chrysopterygius* Gould 1857 is an Endangered species that is only found on Cape York Peninsula, Australia [19]. It has disappeared from 95% of its distribution since 1845, initially from overgrazed floodplain grasslands in the late 19^th^ century [20]. The initial decline has been attributed to a loss of food plants and degradation of the termite mounds in which it nests [20]. In addition, its habitat has undergone encroachment, notably by broad-leaved tea trees (*Melaleuca viridiflora*), for at least the last 100 years [21,22]. All of the habitats considered critical to the parrot’s survival are prone to encroachment (S1 Table). Crowley *et al.* [23] reported that woody plant encroachment is associated with both failure of golden-shouldered parrot nests and predation of attending adults. Current management to restore open nesting habitat by reducing grazing pressure, storm-burning and clearing dense vegetation around nests is predicated on reversing these impacts [23–25]. However, largely based on three unsuccessful predation events in open vegetation, Collingwood *et al.* [26] concluded that golden-shouldered parrot nest success improves with woody plant density. Clarifying whether this is the case is therefore an imperative for golden-shouldered parrot recovery.

**Fig 1:**
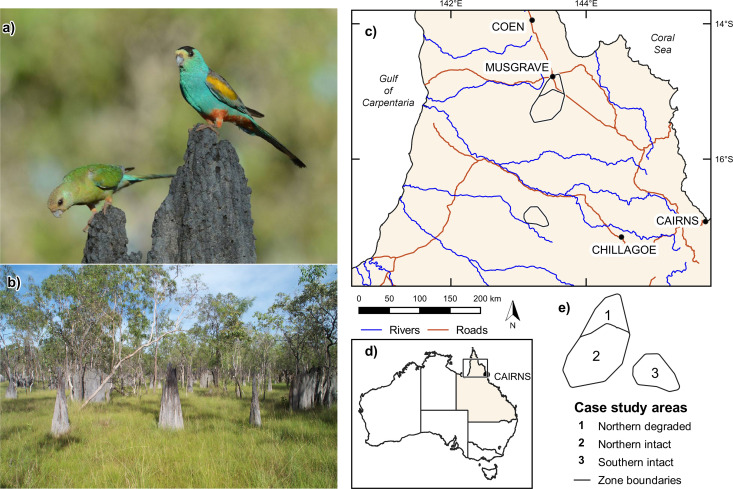
Golden-shouldered parrots, their nesting habitat, and the study area. (a) Female (left) and male parrots on a nesting mound built by *Amitermes scopulus*. (b) Partially occluded nesting habitat with *A. scopulus* mounds in the foreground and *A. laurensis* mounds in the background. Location of study area in relation to (c) Cape York Peninsula, and (d) Australia. (e) Nesting zones. Photo credits: (a) Peter Valentine, (b) Gabriel M. Crowley.

A small, sexually dimorphic bird (Fig 1a), the golden-shouldered parrot nests in mounds constructed by *Amitermes scopulus* (conical mounds), *A. laurensis* (magnetic mounds), *A. vitiosus* (columnar mounds) and *Nasutitermes triodeae* (boulder mounds) along – or adjacent to – seasonally-flooded, grassy drainage lines (Fig 1b). It has a commensal relationship with the coprophagous Endangered antbed parrot moth *Trisyntopa scatophaga*, which keeps the parrot’s nests clean by consuming their faeces [27].

The parrots feed on grass seeds, supplemented by legumes when breeding, and a range of other foods when grass seeds become scarce in the early wet season [23]. Nesting is initiated on the seeding of cockatoo grass *Alloteropsis semialata* and herbaceous legumes [23]. Mortality is thought to be driven primarily by predation during the breeding season and during a period of early wet season food shortage [19]. The adults, particularly the females, are vulnerable to predation when excavating or attending the nest [27]. Females undertake most nest excavation and all egg incubation, during which males provide additional predator awareness [27]. Predator awareness is also enhanced through the parrot’s association with mixed-species flocks whose focal species is the black-faced woodswallow *Artamus cinereus* [27,28].

In order to clarify the effects of encroachment on golden-shouldered parrots, we analyse the impact of woody plant density on golden-shouldered parrot nesting at two scales by first analysing in detail the nest outcome data that were referenced in Crowley *et al.* [23]; and then assessing the impact of vegetation structure and change on nesting activity across the species’ contemporary breeding range. To inform this analysis, we also assess whether golden-shouldered parrots preferentially nest in open vegetation; measure the extent to which encroachment is occurring in the parrot’s distribution; and examine the feedback loop between encroachment and fire regime [29].

## Methods

### Study area and periods

The study area covered the species’ recent distribution (140°–145° E and 12°–19° S) on Cape York Peninsula, Australia (Fig 1) [19]. It comprised two discrete areas: the Northern nesting area (2,634 km^2^), south of Musgrave; and the Southern nesting area (843 km^2^); west of Chillagoe (Fig 1). The region has a tropical savanna climate and annual rainfall between 800 and 1,500 mm [30]. *Eucalyptus*/*Corymbia* woodland predominates on extensive residual sands and weathered surfaces; and *Melaleuca* woodland and grassland on the interspersed alluvial fans and channel deposits [31,32]. The earliest evidence of occupation by First Peoples is from 37,000 years BCE [33]. Olkola, Kunjen and Thaypan Peoples are the Traditional Owners of the Northern nesting area; and Kokoberrin and Wakaman Peoples of the Southern nesting area [34]. From the 1870s, they were progressively displaced as cattle grazing became the dominant land use across the majority of the parrot’s distribution [20]. Between 1996 and 2022, about 4.2 million ha of Cape York Peninsula was returned to the Traditional Owners [35]; in 2014, 63% of the Northern nesting area was returned to the Olkola People and 7.5% to the Thaypan People.

The study area was further subdivided into the Northern degraded zone (in which grazing intensity had increased through the study period and the number of parrots nests had severely declined from 2009 [34], except in a small area that has since been under targeted conservation management for the parrots [25]), and the Northern intact and Southern intact zones (neither of which had been heavily grazed or developed, and nor showed evidence of a decline in parrot nesting activity between 1990 and 2020). Feral cattle, pigs and horses occur throughout the study area, but have been subject to ongoing control programs [36,37].

Nesting was monitored in the Northern nesting area over the 1993–1997 golden-shouldered parrot breeding seasons (May–August). Change in vegetation structure between 1990 and 2020 and fire frequency between 2000 and 2020 were measured in the four nesting zones.

### Data collection

#### Nest locations.

In 1992, STG had searched all drainage lines in grassland and open woodland in the study area on foot or motorbike to locate termite mounds of a suitable size for nesting (> 1 m high and > 30 cm diameter at the ground). These mounds were revisited in the breeding seasons (March–July) of 1993–1997 to identify nests, which were readily identifiable as round holes ~4.5 cm in diameter leading into a straight tunnel, and could be seen from ~30 m away in open vegetation. We used a different subset of the nests in each analysis, depending on the data collected (Table 1). To minimise the chance of repeat sampling of nests of the same adults or the same nesting territory, no nest was included in the S1 or S2 Data if it was built in the same mound as – or was attended by a banded, or otherwise distinctive, bird that had previously attended – a nest that was already in the dataset. Further (as nests of different pairs may be as close as 100 m, but are never in sight of each other [27]), we excluded any nest that was within 100 m of a nest already in a dataset in that year, or within 50 m of any other nest already in the dataset.

**Table 1:** Number of nests used in this study for assessment of nesting outcomes of golden-shouldered parrot on Cape York Peninsula.

Dataset	Year					Total	Study	Models	Description
	1993	1994	1995	1996	1997				
S1 Data.	13	48	–	–	–	61	Nest site location	Negative-binomial generalised linear mixed models WPD1–WPD4	Stem density ~ quadrat location and stem size class
“	“	“	“	“	“	“	Bitterlich calibration	Stepwise regression models BITT1 and BITT2	Bitterlich score ~ quadrat location and stem size class
S2 Data.	13	40 (3)	55 (8)	–	–	108 (11)	Nest outcomes	Descriptive statistics only	Termite mound use, rates of egg laying, hatching and fledging, causes of nest failure, and predation rates.
“	“	“	“	“	“	“	Impact of vegetation structure	Asymptotic linear-by-linear association tests	Fate of offspring ~ Bitterlich class Loss at different life stages ~ Bitterlich class Predation rates (by reptiles, by butcherbirds, total) ~ Bitterlich class
“	“	“	“	“	“	“	“	Logistic model AF1, logistic exposure models AF2–AF5	Any fledging ~ Bitterlich score with or without location, week and/or year
“	“	“	“	“	“	“	“	Logistic model TNS1, logistic exposure models TNS2–TNS5	Total Nest Success ~ Bitterlich score with or without location, week and/or year
S3 Data.	3	13	18	43	27	104	Other causes of nest failure	Descriptive statistics only	Causes of nest failure and predation rates.

Numbers in brackets indicate nests with cameras.

#### Vegetation structure.

We used Bitterlich score to assess the effect of vegetation structure around nests as it prioritises the closest over more distant stems [38], and therefore samples those most likely to influence nest outcomes. We also measured plot-based stem densities at 61 nests (S1 Data) in a grid of 16 10 m x 10 m quadrats centred on each nest to provide comparison to Collingwood *et al.* [26]; calibrate Bitterlich score against stem density; and assess whether stem density influences nest location. We counted the number of live and dead woody stems in three size classes: small (2–5 cm diameter at breast height; DBH), medium (5–10 cm DBH) and large (> 10 cm DBH) in four inner quadrats (close to the nest) and 12 outer quadrats (surrounding vegetation). Stem counts in inner quadrats were multiplied by three to retain values as integers, while accounting for differences in number of quadrats sampled.

#### Nest outcomes.

Nest outcomes – and the influence of Bitterlich score on them – were recorded at 108 nests (S2 Data) in the Northern nesting area in the 1993–1995 breeding seasons (March–June). All nests were monitored at regular intervals (usually every 3–5 days), covering a total nesting period of 3,113 days. The following details were recorded: location, Bitterlich score, termite mound type; period of time nesting activity was under observation [39]; number of eggs that were laid and hatched; number of chicks that fledged; losses of eggs, chicks and adults; and causes of losses. We assumed that a chick had fledged successfully if it was no longer in the nest ~five weeks after hatching, but had been present the previous time the nest had been observed, and that antbed parrot moths had pupated in the nest wall, as this only occurs if the chicks successfully fledge [40]. Where skeletal remains or flight feathers indicated that an adult bird had been killed at the nest, the nest was watched closely for the next 2–3 days to determine which, if any, adults were attending the nest. We also recorded year and week that the first egg was laid, when necessary, back-calculating this variable based on an egg period of 21 days and estimated chick age [27]. Eleven of the nests were further monitored using motion-activated video cameras placed 3–4 m from the nest entrance, totalling 179 days of observation.

We also recorded causes of egg, chick and adult loss for an additional 104 nests from the 1993–1997 breeding seasons (S3 Data) for which the outcome was known, but did not meet the selection criteria for inclusion in S2 Data, or lacked vegetation data; and made opportunistic observations of interactions between the parrots and potential predators throughout the breeding season, both at and away from nests, as well as interactions recorded on camera.

#### Landscape-scale habitat loss.

To examine the landscape-scale impact of encroachment on the golden-shouldered parrot and the process of encroachment, we assessed foliage cover around known nest sites in each nesting zone in 1990 and 2020 and fire frequency between 2000 and 2020. Foliage cover was derived from Autumn Persistent Green [APG, measured between March and May; 41]. APG is a satellite-derived measure of canopy foliage, which is based on the green fraction in Landsat imagery that persists through the year [42]. Fire frequency (number of fires between 2000 and 2020) was also derived from satellite imagery, but not available before 2000 [43]. We used QGIS [44] to calculate averages for each measurement for a 100 m buffer around each of 570 nests (S4 Data). To minimise the effects of spatial autocorrelation, these nests had been spatially thinned from a dataset of 1,460 nests found in surveys between 1977 and 2017 using QGIS [44]. As encroachment on Cape York Peninsula is associated with particular vegetation types [22], we also extracted vegetation type [1:5 M Broad Vegetation Group; 45] for each nest buffer.

### Statistical analysis

All analysis was undertaken in R [46], and the significance threshold for all tests was P < 0.05. Assumptions of all analyses were examined, and – where required – adjustments made to ensure these were met, as follows. Independence between nests was assumed because of the data collection methods. Further, spatial autocorrelation was incorporated into models using the S2 Data, in which average nearest neighbour distances between nests (527 m ± 52 SE) were lower than they were for the S1 Data (917 m ± 118 SE). Homogeneity of variance was tested using Bartlett’s K^2^ [47]. Linearity was assessed by examining plots of the predictors and the log-odds of the response variable for logistic regressions models [48], and between the residuals and fitted values for all other regression models [49]. Multicollinearity was assessed using the variance inflation factor [49]. Models with extreme outliers were re-run without them to assess their likely impact. Over- and under-dispersion of negative binomial models were tested using the R package performance [50].

#### Stem density.

To assess whether golden-shouldered parrot nest locations are influenced by woody plant density, we used generalized linear mixed models fit by maximum likelihood [51] for negative binomial distributions using the S1 Data. Stem density was the dependent variable, and quadrat location and stem size class the independent variables; nest identification number was included as a random effect. We repeated the models with and without interactions, and with and without extreme residuals. We selected the best fit model based on lowest values of Akaike Information Criterion (AIC), log-likelihood, deviance, and lastly Bayesian Information Criterion [51].

#### Bitterlich calibration.

To calibrate Bitterlich score against the density of stems in each size class and quadrat combination, we used stepwise regression for a negative binomial distribution (S1 Data). The best-fit model was selected based on the lowest AIC [52].

#### Impact of vegetation structure.

To test the influence of vegetation structure on categorical nest outcomes, we divided the S2 Data into three quantile Bitterlich size classes and compared them using asymptotic linear-by-linear association tests [a form of χ2 test that accounts for ordinal data; 53]. We also modelled the influence of Bitterlich score on the binary outcomes of Any Fledging (nest did/did not produce at least one fledgling) and Total Nest Success (all observed fertile eggs did/did not produce fledglings) using first uncorrected logistic regression, and then logistic exposure regression [which corrects for the time each nest was under observation; 54]. We re-ran the exposure models to assess whether the addition of spatial autocorrelation (using generalised additive modelling), week, or year [as a random effect using glmer from the R package lme4; [51] improved model fit, based on residual deviance [55], Tjur’s R^2^ [coefficient of discrimination; [56] and model fit and Bitterlich fit [how well the dependent variable was predicted by the model and Bitterlich score, respectively; [55] using the binnedplot function (with 10 bins) from the R package arm [57].

#### Landscape-scale habitat loss.

We compared 1990 and 2020 foliage cover and fire frequency (2000–2020) between nest zones using Kruskal-Wallis (KW) rank sum tests because of significant variance differences (S4 Data). Dunn’s test with Benjamini-Hochberg (BH) adjustment was used to identify significant pairwise differences between zones.

To examine the feedback loop between encroachment and fire regime in the parrot’s contracting distribution, we used generalised additive modelling [GAM; with factor smooth from the mgcv R package; 58]. We first assessed the influence of vegetation type and initial foliage cover on subsequent fire frequency (Model FF1); and then assessed the influence of vegetation type, initial foliage cover and fire frequency on the change in foliage cover between 1990 and 2020 (Model FCC1). gam.check was used to assess model fit, accepting models with a k-index that indicated no significant patterns in the residuals at P < 0.05 [59].

## Results

### Nest site location

**Fig 2:**
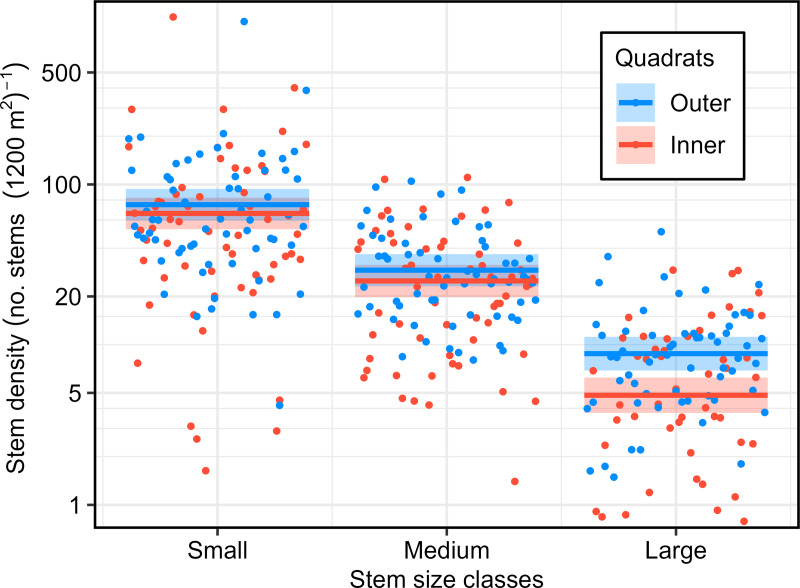
Influence of quadrat location and stem size class on stem densities around golden-shouldered parrot nests. Inner quadrats = four 10 m by 10 m quadrats cornering each nest. Outer quadrats = 12 10 m by 10 m quadrats surrounding the inner quadrats. Horizontal lines are responses estimated by the model. Points are partial residuals. Ribbons are 95% confidence intervals. See S2 Table for model parameters (Model WPD1). Sample size = 61 (S1 Data).

Woody plant densities around golden-shouldered parrot nests (S1 Data) averaged 10.2 (± 1.1 SE) stems per 100 m^2^ in the inner four quadrats, and 11.2 (± 1.1 SE) stems per 100 m^2^ in the outer 12 quadrats, but were not normally distributed. The best-fit model (Model WPD1; S2 Table) indicated that outer quadrats had significantly more stems than inner quadrats (P < 0.001), and that the number of stems decreased with diameter size class, with a predominantly linear trend (P < 0.0001). It estimated that inner quadrats had 11.8% fewer small stems, 14.4% fewer medium stems, and 45.0% fewer large stems than outer quadrats (Fig 2). Removal of two extreme outliers only marginally changed the estimates, and not the significance levels of any term.

### Bitterlich calibration

The best-fit model explaining the relationship between Bitterlich score and stem counts (Model BITT2) indicated that Bitterlich score predominantly reflected the number of medium stems in the inner quadrats (Z = 4.02, P < 0.0001), with the number of small stems in the inner quadrats being significant but less influential (Z = 2.05, P = 0.0356; S3 Table).

### Categorical nest outcomes

Of the 108 nests monitored for both nest success and Bitterlich score (S2 Data), 36.1% were in the Northern degraded zone and the remainder in the Northern intact zone; 3.7% were in magnetic mounds and the remainder in conical mounds. Median period of nest observation was 29 days. The first egg was laid in the 9^th^ week of the year (26 February–5 March). Median and mean week of first egg-laying was week 14 (2–4 April); and the latest nest started in week 23 (4–11 June). In total, these nests produced 555 eggs, 74.6% of which hatched, and 50.6% of which fledged; 67.9% of hatchlings fledged. Of these nests, 79.6% produced at least one hatchling, 62.0% produced at least one fledgling, and 32.4% produced fledglings from every fertile egg observed. Nest outcomes were bimodal: 38.0% of nests completely failed, 9.3% produced one or two fledglings, and 52.8% of nests produced between three and six fledglings. Of the 86 nests in which one or more eggs hatched, Any Fledging occurred in 77.9%; and Total Nest Success occurred in 40.7%. The number of fledglings produced per nest averaged 2.6 (± 0.23 SE). The 67 nests in which fledging occurred produced an average of 4.2 (± 0.18 SE) fledglings. Of the 11 nests that had cameras, nine produced a total of 33 fledglings, and two produced none. Fledging was not captured on video. We personally witnessed fledging at two of the 108 nests in our vegetation dataset (one with an operating camera) and at 16 nests in total.

### Causes of nest failure

We estimate that predation of either adults or offspring occurred at between 23.1% (based on direct evidence) and 58.3% (also including unexplained disappearance) of nests (S4 Table; S2 Data). At least one adult disappeared or was killed at 33.3% of nests, with remains of the birds being found in half of these. When one parent was killed, the remaining adult continued to rear the chicks until fledging. Within a couple of days of a male being lost, the female paired with another male who did not feed the chicks. At one nest, both the initial and replacement males were lost.

At 16.7% of nests, we found dead nestlings (8.3%) or adults (13.0%) with damage that was consistent with a butcherbird attack. Both pied *Cracticus nigrogularis* and black-backed *C. mentalis* butcherbirds were seen at nests; the former being filmed at four nests. We also frequently witnessed pied butcherbirds attacking golden-shouldered parrots feeding on the ground. Butcherbird presence did not always result in nest losses: we witnessed parrots successfully chasing a black-backed butcherbird from a nest entrance. However, damage consistent with butcherbird attack was found at several nests, including cracked skulls at 11 nests, and carcass remains wedged into the top of one nesting mound. Of the 18 nests that appeared to be attacked by butcherbirds, ten produced a partial clutch of fledglings, and two produced a full clutch of fledglings.

Reptile predation was assessed as likely at 7.4% of nests and possible at an additional 22.2% of nests. A black-headed monitor *Varanus tristis* eating a chick was filmed at one nest, and large scratch marks consistent with yellow-spotted monitor *V. panoptes* were found at another nest. None of the 33 nests that appeared to have been attacked by a reptile produced fledglings. Predation by an unknown predator was also deemed possible at 13.0% of nests, and we saw a blue-winged kookaburra *Dacelo leachii* take a juvenile golden-shouldered parrot after it had left the nest.

Other causes of nest losses recorded at these 108 nests (S2 Data) included egg infertility (13 nests); fertile eggs that failed to hatch (6); nestling death from unknown causes (10); eggs being scraped out (possibly by an adult parrot preparing for a new clutch (1 nest); and moths pupating at the entrance preventing fledging (1). Four nests were infested with mites *Ornithonyssus bursa* and one contained meat ants *Iridomyrmex purpureus*, but presence of either species did not always prevent fledging. Nest losses in the additional 104 nests monitored (S3 Data; 41.3% in Northern degraded zone; 58.7% in Northen intact zone) were attributed to abandonment (18 nests), predation by reptiles (12), predation by butcherbirds (8), eggs being scraped out (7), and termites fixing the eggs to the floor (7). Six nests contained mites and two contained meat ants. In the one nest that lacked moth larvae, faeces and mud caking the parrots feet preventing fledging.

### Impact of vegetation structure

#### Categorical outcomes.

**Fig 3:**
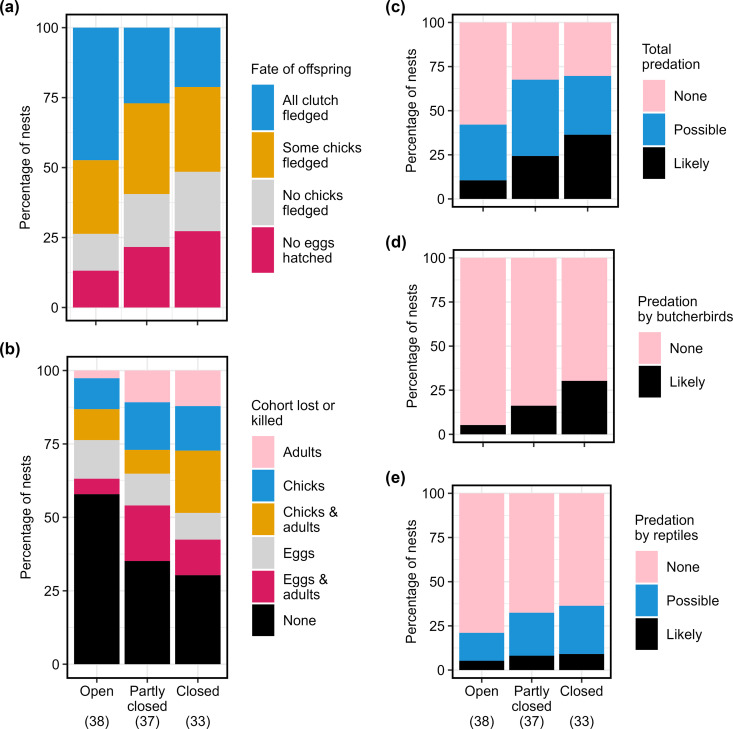
Influence of vegetation density on outcomes of golden-shouldered parrot nests. (a) Fate of offspring to fledging; (b) rate of offspring loss through disappearance or predation; and likely and possible predation rates: (c) total predation; (d) predation by butcherbirds; and (e) predation by reptiles. Bitterlich scores: Open = 0–3, Partly-closed = 4–7, Closed = 8–20. Sample size = 108 (S2 Data).

An asymptotic linear-by-linear association test showed that measures of nest success decreased significantly as vegetation density increased (Z = 2.36, *χ*^2^ = 5.58, P = 0.0182; Fig 3a). The loss of eggs, chicks and/or adults from nests also increased significantly with vegetation density (Z = −2.31, *χ*^2^ = 5.32, P = 0.0211; Fig 3b). Adults disappeared from 18.4% of nests in open vegetation, and 45.5% of nests in dense vegetation. Predation rates across all life stages also increased significantly with vegetation density (Z = −2.88, *χ*^2^ = 8.30, P = 0.0040; Fig 3c). Of the four nests at which butcherbirds were filmed, predation was unsuccessful in the two nests in open vegetation, and appears to have been successful in each of the nests in closed and partly closed vegetation. An increase in predation by butcherbirds from 5.3% in open vegetation to 30.3% in closed vegetation was also significant (Z = −2.80, *χ*^2^ = 7.87, P = 0.0050; Fig 3d). However, an increase in losses attributed to predation by reptiles from 21% to 32% once Bitterlich score exceeded three was not significant (Z = −0.51, *χ*^2^ = 0.26, P = 0.6101, Fig 3e).

#### Nest success.

**Fig 4:**
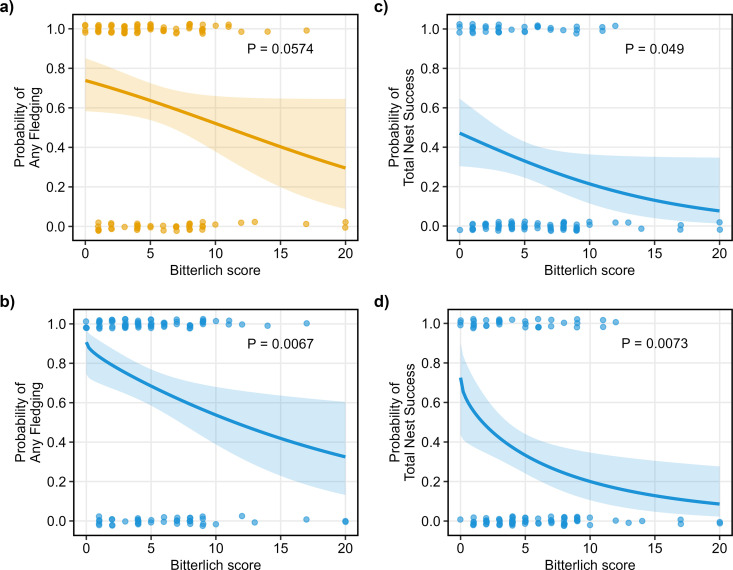
Influence of vegetation density on nest outcomes. Influence of Bitterlich score on Any Fledging: (a) simple logistic regression; and (b) best-performing logistic exposure regression (AF2). Influence of Bitterlich score on Total Nest Success: (c) simple logistic regression; and (d) best-performing logistic exposure regression accounting for week of laying (TNS4). Days of observation and week were set at the median values of 29 and 14, respectively. Plots with significant P values are presented in blue. Sample size = 108 (S2 Data).

Any Fledging rate decreased with vegetation density (Fig 3a). The simple logistic regression model (Model AF1) failed to identify this relationship as significant (S5 Table, Fig 4a). However, all logistic exposure models (models accounting for observation period) did identify a significant negative effect of Bitterlich score on Any Fledging. AF2 (which included Bitterlich as the only independent variable) was selected as the best-fit exposure model based on model and Bitterlich fit (S1 Fig), and the failure of additional terms to significantly reduce residual deviance (S5 Table). It predicted that at least one fledgling would have been produced in 90.7% of nests when the Bitterlich score was zero, declining significantly to 32.5% at a score of 20. Half of this reduction occurred as Bitterlich score increased from zero to eight.

Total Nest Success decreased with vegetation density (Fig 3a). The simple logistic model (Fig 4c) and all logistic exposure models recognised this relationship as significant (S5 Table). TNS4 (which included Bitterlich and week as independent variables) was selected as the best-performing model, based on model and Bitterlich fit (S2 Fig), even though the inclusion of week did not significantly improve residual deviance (S5 Table). It predicted that Total Nest Success would be 72.3% when the Bitterlich score was zero, and decline significantly to 8.5% at a score of 20. Half of this reduction was experienced as Bitterlich score increased from zero to five.

### Landscape-scale habitat loss

**Fig 5:**
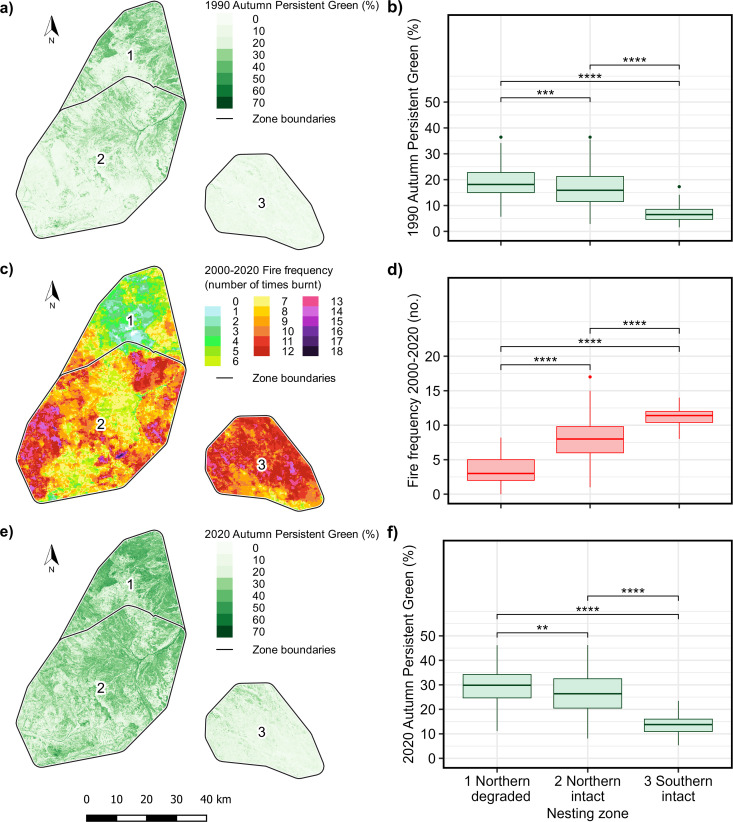
Comparison of foliage cover and fire frequencies in golden-shouldered parrot nesting zones. Autumn Persistent Green in 1990: (a) in nesting zones, and (b) averaged across 100 m buffers around individual nests. Fire frequency between 2000 and 2020 (c) in nesting zones, and (d) averaged across 100 m buffers around individual nests. Autumn Persistent Green in 2020 (e) across nesting zones, and (f) for 100 m buffers around individual nests. Number of nests: Northern degraded, 177; Northern intact, 319; Southern intact, 74. Probabilities for pairwise significant differences indicated on plots: ***, P < 0.001; ****, P < 0.0001. Data sources: Autumn Persistent Green: Department of Environment and Science [60]; Fire Frequency: Charles Darwin University [43]. Sample size = 570 (S4 Data).

In 1990, foliage cover varied significantly between the nesting zones (KW *χ*^2^ = 157.89, N = 570, DF 2, P < 0.0001). It was highest in the Northern degraded zone, and lowest in the Southern intact zone (Fig 5a, b). Fire frequency between 2000 and 2020 varied significantly between the nesting zones (KW *χ*^2^ = 164.5, N = 570, DF 2, P < 0.0001). It was lowest in the Northern degraded zones and highest in the Southern intact zone (Fig 5c, d). By 2020, foliage cover had increased across all nesting areas. It varied significantly between the nesting zones (KW *χ*^2^ = 303.8, N = 570, DF 2, P < 0.0001). Ranking of the zones did not change between 1990 and 2020, but the magnitude of the difference between the two Northern zones had decreased (Fig 5e, f). Dunn’s test indicated that all differences between all zones were significant at BH-adjusted P < 0.05 (S6 Table).

GAM (Model FF1) identified a significant impact of both vegetation type (S7 Table) and initial foliage cover (P < 0.0001) on subsequent fire frequency, and explained 31.4% of the residual deviance. FF1 was accepted as a robust model based on residuals and k-index. Three fire frequency responses associated with vegetation types could be identified from this model (S3 Fig), increasing significantly from grassland through eucalypt woodland and floodplain forest to tea tree woodland and heath. The model predicted that fire frequency would be reduced by 40% as initial foliage cover increased from 2% to 17%, and that further increases in initial foliage cover had minimal influence on fire frequency.

GAM (Model FCC1) identified significant impacts of vegetation type (S4 Fig), initial foliage cover (P < 0.0001) and fire frequency (P = 0.0203) on subsequent change in foliage cover, and explained 12.7% of the residual deviance. FCC1 was accepted as a robust model based on the same criteria. It identified three foliage cover responses associated with vegetation types (S4 Fig), increasing significantly from floodplain forest through eucalypt woodland, tea tree woodland and grassland to heath. The model predicted that increase in foliage cover peaked in vegetation that had an initial foliage cover of 19%; and that the effect of fire on foliage cover change peaked at 0–5 fires in 21 years, then decreased, levelling off at about 12 fires in 21 years.

## Discussion

This study found that woody plant encroachment adversely affected golden-shouldered parrots, as follows. Failure of eggs to hatch and chicks to fledge increased with woody stem density, with this effect becoming evident at relatively low densities. Both total predation and predation by butcherbirds increased with woody stem density. Woody plant encroachment adversely affected both measures of nest success. The contracting section of the parrot’s distribution had the highest initial foliage cover around nests, and the greatest increase over the three-decade study period. Possibly as a result of these impacts, the parrots preferentially nested in areas that were significantly more open than the surrounding habitat. These results are discussed in more detail below.

### Drivers of woody plant encroachment

The current study supports previous work showing golden-shouldered parrot’s grassland habitat on Cape York Peninsula, Australia, has been progressively invaded by woody plants [21,22], and that suppression of fire regime amplifies encroachment, whereby fires become less frequent as woody vegetation cover increases, and encroachment becomes more likely as fire frequency declines [29,61]. The main driver of this process appears to be cattle grazing [22], which removes the grasses that suppress recruitment of seedlings and suckers to the canopy [62], and reduces the biomass needed to fuel the fires that keep the suckers below the top-kill zone [63,64]. Removal of First Nations Peoples from the time of the first cattle stations, and prohibition of their traditional burning practices where they remained [65], further promoted woody thickening [21]. Similar processes have been responsible for woody invasion of grasslands across the tropical world [3]. This study also demonstrates that, once encroachment has reached threshold levels, fire is no longer effective, and mechanical and/or chemical intervention will be required to reverse it [25].

### Impact of encroachment

Woody plant encroachment may reduce nesting success through increased predation (e.g., songbirds in Mediterranean shrubland [15] and North American prairies [66]), and/or through reduced access to food resources (e.g., raptors of open country in African savannas [67]). Although predation of golden-shouldered parrots increased with encroachment, this relationship did not explain all increases in nest failure. Therefore, both predation and food limitation may be in operation. Brood parasitism has also been found to increase with encroachment in North American prairies [68], but is not an issue for parrots [69].

The failure of all attempts at predation on golden-shouldered parrots in open vegetation that have been captured on camera (two in this study and three by Collingwood *et al.* [26]) illustrates the influence vegetation structure has on predation success. Lack of perches near the nest would disadvantage ambush predators, such as butcherbirds [70], while open vegetation would allow the parrots ample time to detect predation attempts. It therefore appears that, as vegetation thickens, losses to predation will increase as the abundance of woodland predators increases, and the success of predation attempts by both butcherbird species increases, irrespective of any change in their abundance.

Adverse effects of encroachment were evident at all parrot life stages. Our analyses show that high levels of fledging in treeless vegetation sharply declines with the presence of only a few trees. As encroachment occurs at a landscape-scale, adults foraging away from the nest may also experience increased predation pressure. The energetic cost of foraging will also increase if the parrots are forced to fly further afield because the grasses they feed on are competitively replaced as woody cover increases [71]. One of the parrot’s most important food plants, cockatoo grass, is also sensitive to grazing [72] and declines in the absence of fire [73], so may be eliminated by the same processes that drive encroachment, as well as because of it. Decreased food availability and increased predation risk also interact, with birds being more exposed to predators as the period needed to find food increases, and becoming less efficient at gathering food as the need to be vigilant for predators increases [28,74,75]. To minimise predation risk when feeding, the parrots – like their congener, the hooded parrot *Psephotellus dissimilis* – associate with mixed species flocks [28]. The focal species of these flocks, the black-faced woodswallow, has also declined on Cape York Peninsula [76], and also appears to rely on an open vegetation structure [77]. Hence, encroachment is likely to increase predation at and away from the nest through many pathways.

Several species of north American prairies, African savannas and European alpine grasslands [6,10–13,15,16,18,67,78–87] have a similarly negative response to encroachment of woody vegetation. These species often have well-camouflaged eggs and dorsal plumage, or nest under the cover of grasses. In contrast, the nesting success of species that require concealment from predators (e.g., sage grouse *Centrocercus urophasianus* [88], clay-colored sparrows *Spizella pallida* and vesper sparrow *Pooecetes gramineus* [89] in North American prairies) improves with proximity to woody vegetation. The response to encroachment will also be affected by a shift in the dominant predators between woodland and grassland [81].

Our findings support the earlier conclusion of Crowley *et al.* [23] that encroachment increases predation at golden-shouldered parrot nests and reduces nest success, but contradict that of Collingwood *et al.* [26, p 168], who stated that “Nest success was higher amongst denser vegetation, indicating that cover may inhibit detection of nests by predators, particularly at fledging time”. This discrepancy may have arisen from the latter’s failure to account for observation period. Even in our study, the extent of the effect of encroachment on fledging was obscured before we accounted for observation period. This is because – while nests in the open can be seen from some distance – detection of nests in dense vegetation is more reliant on bird activity, which increases through the breeding season. Hence, nests in dense vegetation that fail early in the breeding season are likely to be under-sampled [39,54,90]. Other factors contributing to the discrepancy were the fact that the study by Collingwood *et al.* [26] was undertaken in relatively open vegetation (mean stem densities being between one-quarter and one-eighth of those in the current study); and it included failed predation attempts as actual predation events – even though such attempts are unlikely to prevent successful fledging.

### Nest site selection

Our results suggest that golden-shouldered parrots select nests sites in the most open areas available because this is where they are most likely to survive the nesting period and produce most young. Similar selectivity has been observed in Henslow’s sparrow, eastern meadowlark *Sturnella magna*, and bobolink *Dolichonyx oryzivorus* in North American prairies [81]. In the golden-shouldered parrot, selective nesting in open vegetation could also be an artefact of the availability of suitable termite mounds, which can be damaged by cattle and pigs [91]. The mounds may also be negatively affected by changes in insolation, grass cover, and soil fertility and acidity [92,93] that are associated with encroachment, as *Amitermes* spp. feed on grass and are sensitive to shading [94,95]. Hence, the processes that drive encroachment may also eliminate suitable nesting mounds. Without further study, it is therefore not possible to tell whether the parrots made an active choice to nest in open areas, or whether they are restricted to doing so by the limited availability of suitable mounds.

### A true grassland species

Species that are least vulnerable to predation are generally inconspicuous, having eggs and females that blend in with their environment [96]. Mound-nesting distinguishes the golden-shouldered parrot (along with the hooded parrot and the extinct paradise parrot *P. pulcherrimus*) from the more-ancestral, congeneric mulga parrot, *P. varius* [97], which nests in trees [27]. As in other cavity-nesting birds [98], mound-nesting enabled the parrots to nest away from arboreal predators while still concealing their white eggs [27]; and the female’s green plumage blends in with the verdant grasses that predominate in the wet season/early-dry season (Fig 1a, b), when the parrots nest. Furthermore, the golden-shouldered parrot’s use of magnetic mounds allowed it to move into seasonally flooded open grassland [20]. This transition probably began after the species’ separation from the hooded parrot around 7 million years ago [97], coinciding with the development of the extensive grassy floodplains of southwestern Cape York Peninsula [99]. The end result is that the golden-shouldered parrot is a grassland species of woodland origin that is now highly susceptible to woodland predators.

### Population decline and contraction

Our study covered a period of golden-shouldered parrot population decline. This was evident in the abandonment of most nesting territories in the northern degraded zone (which contained ~40% of the nests monitored in this study) by 2009. Encroachment was already evident in this zone in the early 1990s [21], and our study shows that it continued to progress through to 2020. The unfavourable impact of encroachment on nesting outcomes demonstrated here is likely to have contributed to the contraction of the parrot’s distribution. Moreover, if encroachment also caused the termite mounds to deteriorate (as argued above), it is also possible that finding mounds that are suitable for nesting has become increasingly difficult.

A species will decline if each adult produces fewer than one progeny that survives to breeding age before the adult dies. Our analysis indicates that, in the absence of trees, almost all nests would be expected to produce at least one fledgling. Assuming that the species’ population was stable before encroachment of its grassland habitat, we take this value as an approximation of the nest success required for population replacement. If this is the case, then nest success under the highest stem density we measured would have been around one-third of the putative rate required for population replacement.

The impacts of encroachment on the golden-shouldered parrot would be expected to extend beyond the nesting period, especially where it occurs at the landscape scale. Newly fledged chicks are highly vulnerable to predation [100], particularly in the eight weeks before the parrots can feed themselves independently [27], and again in the early wet season, when they spend extended periods feeding on the ground [23,28] in habitat that is also prone to encroachment (S1 Table). Hence, the differences in survivorship of parrots in open and thickened areas are likely to intensify once the birds leave the nest, potentially explaining the loss of the species from much of the northern extremity of its distribution.

Adult mortality has an even greater impact on population stability. Modelling of other tropical birds shows that an annual adult mortality of 20–25% is likely to cause extinction, whereas mortality of fledglings in their first year has to be as high as 70% to achieve the same effect [101–103]. This suggests that loss of one or more adults from nearly half of the nests found in dense vegetation would be close to that needed to drive the species’ decline. Any additional adult mortality in wet season feeding habitat would drive the parrots closer to extinction, regardless of breeding success.

However, some long-abandoned nesting areas still retain a healthy grassland structure [22], notably in the floodplains of southwestern Cape York Peninsula where European explorers first encountered the parrots, and east of Coen where the first parrot nests were officially recorded [20]. These areas were the first to be heavily grazed, and have lost important food plants [20] – also as a product of grazing and a change in fire regime [72]. Hence, the parrots may have first disappeared from overgrazed floodplain grasslands because of a loss of food plants and degradation of magnetic termite mounds [20]. The impacts of encroachment described here may therefore be a secondary process that has rendered much of the remaining habitat suboptimal by greatly reducing parrot nest success.

### Management

Management adopted to address encroachment of golden-shouldered parrot habitat includes restricting cattle grazing and burning when the plants are actively growing; and tree clearance when encroachment is too advanced to support effective fire management [23–25]. Our results suggest that burning to maintain an open habitat should be undertaken at least every second year [in line with previous recommendations; 63]; and our zone comparisons suggest that this can only be achieved under very light grazing intensity. Such a regime should have multiple benefits for the species, restoring the condition of termite mounds and food plants, as well as reducing predation pressure; and should also benefit a range of co-occurring species that depend on a grassy, open habitat structure, such as the black-faced woodswallow [77] and the painted button-quail *Turnix varius* [104]. As much of the parrot’s habitat has now been retired from cattle grazing with its return to the Traditional Owners [105], it is hoped that destocking and reinstating traditional fire regimes – along with dedicated adaptive management on grazing land [25] – will at least halt the parrot’s decline. Similar management has been recommended in Africa to prevent encroachment causing the extinction of the Archer’s lark [9], and may be more widely applicable. More specifically, it should be considered in the Fly River delta of New Guinea, an area in which invasion of grasslands by *M. viridiflora* is also occurring [106], and is the only known location for the Vulnerable Fly river grassbird *Poodytes albolimbatus* and the black mannikin *Lonchura stygia* [107].

## Conclusions

The golden-shouldered parrot has ancestral woodland origins. Nesting in termite mounds allowed it to evolve to become a grassland species and occupy the vast floodplain grasslands across Cape York Peninsula, Australia. As a result, the species is now highly vulnerable to woodland predators. The introduction of cattle grazing and disruption to traditional fire regimes with the dispossession of the Traditional Owners resulted in the degradation of grasslands, and the contraction of parrots to the refugial areas along narrower drainage lines. Woody plant encroachment in these areas has reduced nest success through increased predation, and probably also increased predation in feeding areas. The parrot has disappeared from properties as grazing pressure increased, fire frequency declined, and encroachment intensified. Restoration of golden-shouldered habitat will require destocking or a reduction in grazing pressure, an increase in fire frequency, and clearance of heavily thickened vegetation. This process has commenced with the return of the majority of the species’ current distribution to the Traditional Owners, and dedicated adaptive management programs on the areas that are still grazed.

## Supporting information

S1 Data Woody stem density and Bitterlich score for vegetation around 61 golden-shouldered parrot nests. (XLSX)

S2 Data Nest outcomes and Bitterlich score for 108 golden-shouldered parrot nests. (XLSX)

S3 Data Nest outcomes for an additional 104 golden-shouldered parrot nests. (XLSX)

S4 Data Foliage Cover, Fire Frequency and Vegetation Type in 100 m buffers around 570 golden-shouldered parrot nests in three nesting zones. (XLSX)

S1 Table Habitats used by golden-shouldered parrot during the breeding and wet seasons, and their propensity for woody plant encroachment. (PDF)

S2 Table Generalised linear mixed models comparing woody plants densities of three size classes in inner and outer quadrats around golden-shouldered parrot nests. (PDF)

S3 Table Negative binomial models explaining the relationship between Bitterlich score and number of stems around golden-shouldered parrot nests. (PDF)

S4 Table Estimated predation rates affecting golden-shouldered parrot nests. (PDF)

S5 Table Logistic models examining the influence of vegetation structure and nest outcomes around golden-shouldered parrot nests. (PDF)

S6 Table Dunn’s pairwise comparisons between nesting zones in relation to foliage cover and fire frequency. (PDF)

S7 Table Generalised additive model explaining the influence of vegetation type and initial foliage cover on subsequent fire frequency. (PDF)

S8 Table Generalised additive model explaining influence of vegetation type, initial foliage cover and fire frequency on subsequent change in foliage cover. (PDF)

S1 Fig Assessment of fit for models examining the relationship between Any Fledging and Bitterlich score. (TIF)

S2 Fig Assessment of fit for models examining the relationship between Total Nest Success and Bitterlich score. (TIF)

S3 Fig Predicted outcomes from generalised additive model explaining influence of Vegetation Type and initial Foliage Cover on subsequent Fire Frequency. (TIF)

S4 Fig Predicted outcomes from generalised additive model explaining influence of vegetation type, initial foliage cover and fire frequency on subsequent change in foliage cover. (TIF)
