# Supplementary material for: Woody plant encroachment drives the decline of a grassland bird: The fate of golden-shouldered parrot (Psephotellus chrysopterygius) nests
Source: PLoS One. 2025 Jul 23;20(7):e0327543. doi: 10.1371/journal.pone.0327543 (PMC12286340; doi:10.1371/journal.pone.0327543)
Supplement: S1 Table — (PDF) [file pone.0327543.s005.pdf]

S1 Table. Habitats used by golden-shouldered parrot during the breeding and wet seasons, and their propensity for woody plant encroachment.

		Feeding		
**Habitat**	**Nesting**	**Breeding season**	**Wet season**	**Prone to woody plant encroachment**
Gravel slopes	++		++	Yes
Narrow flats	++		+	Yes
Flat edges	++			Yes
Broad flats	+	+	+	
Box flats	+	+		
Sand ridges and low hills	+	+	+	
Glimmer grass flats			++	Yes
Rocky hills			+	
Bare areas			+	

Legend: Habitat: +: Critical habitat: ++. Based on Crowley GM, Garnett ST, Shephard S. Management Guidelines for Golden-shouldered Parrot Conservation. Cairns: Queensland Parks and Wildlife Service; 2004.
