# Supplementary material for: Woody plant encroachment drives the decline of a grassland bird: The fate of golden-shouldered parrot (Psephotellus chrysopterygius) nests
Source: PLoS One. 2025 Jul 23;20(7):e0327543. doi: 10.1371/journal.pone.0327543 (PMC12286340; doi:10.1371/journal.pone.0327543)
Supplement: S2 Table — (PDF) [file pone.0327543.s006.pdf]

Table S2. Generalised linear mixed models comparing woody plants densities of three size classes in inner and outer quadrats around golden-shouldered parrot nests.

ALL NEST SITES

	**Model WPD1:** Stem no. ~ Quadrats * size class + (1|Nest)				**Model WPD2:** Stem no. ~ Quadrats + size class + (1|Nest)			
**Coefficient**	**Estimate**	**SE**	**Z**	**P**	**Estimate**	**SE**	**Z**	**P**
(Intercept)	2.994	0.094	31.93	< 0.0001	3.014	0.093	32.24	< 0.0001
Outer quadrats	0.2930	0.0785	3.73	0.0002	0.2676	0.0788	3.40	0.0007
Stem size (linear)	-1.848	0.101	-18.28	< 0.0001	-1.669	0.072	-23.26	< 0.0001
Stem size (quadratic)	-0.2744	0.0956	-2.87	0.0041	-0.1821	0.0684	-2.66	0.0078
Interactions								
Outer quadrats: stem size (l)	0.3340	0.1369	2.44	0.0147	-	-	-	-
Outer quadrats: stem size (q)	0.1690	0.1323	1.28	0.2014	-	-	-	-

Scaled residuals

**Quantile**	**0%**	**25%**	**50%**	**75%**	**100%**	**0%**	**25%**	**50%**	**75%**	**100%**
Value	-1.433	-0.607	-0.137	0.507	4.866	-1.415	-0.615	-0.160	0.493	5.083

Adjusted generalised variance inflation factor (aGVIF = $GVIF^{(1/(2*Df))}$) (threshold for acceptance of aGVIF < 1.6).

	**GVIF**	**DF**	**aGVIF**	**GVIF**	**DF**	**aGVIF**
Quadrats	1.016	1	1.008	1.007	1	1.004
Size	4.014	2	1.415	1.007	2	1.002
Quadrats x size	4.029	2	1.417			

Model selection statistics

**Statistic**	**AIC**	**BIC**	**logLik**	**Dev**	**DF**	**AIC**	**BIC**	**logLik**	**Dev**	**DF**
Value	3122	3154	-1553	3107	358	3126	3149	-1557	3114	360

Deviance comparisons (WPD1 < WPD2)**:** X^2 = 7.086, DF 2, P = 0.0289

EXCLUDING NEST SITES WITH EXTREME RESIDUALS IN MODELS WPD1 & WPD2

	**Model WPD3:** Stem no. ~ Quadrats * size class + (1|Nest)				**Model WPD 4:** Stem no. ~ Quadrats * size class + (1|Nest)			
**Coefficient**	**Estimate**	**SE**	**Z**	**P**	**Estimate**	**SE**	**Z**	**P**
(Intercept)	2.967	0.095	31.31	< 0.0001	2.987	0.095	31.61	< 0.0001
Outer quadrats	0.2934	0.0761	3.86	0.0001	0.2671	0.0762	3.50	0.0005
Stem size (linear)	-1.768	0.098	-18.12	< 0.0001	-1.598	0.069	-23.12	< 0.0001
Stem size (quadratic)	-0.3088	0.0925	-3.34	0.0008	-0.2170	0.0662	-3.28	0.0010
Interactions								
Outer quadrats: stem size (l)	0.3189	0.1326	2.41	0.0162	-	-	-	-
Outer quadrats: stem size (q)	0.1687	0.1281	1.32	0.1878	-	-	-	-

Scaled residuals

**Quantile**	**0%**	**25%**	**50%**	**75%**	**100%**	**0%**	**25%**	**50%**	**75%**	**100%**
Value	-1.504	-0.635	-0.148	0.548	2.876	-1.485	-0.605	-0.131	0.505	3.110

Adjusted generalised variance inflation factor (aGVIF = $GVIF^{(1/(2*Df))}$) (threshold for acceptance of aGVIF < 1.6).

	**GVIF**	**DF**	**aGVIF**	**GVIF**	**DF**	**aGVIF**
Quadrats	1.019	1	1.009	1.007	1	1.003
Size	4.040	2	1.418	1.007	2	1.002
Quadrats x size	4.059	2	1.419	-	-	-

Model selection statistics

**Statistic**	**AIC**	**BIC**	**logLik**	**Dev**	**DF**	**AIC**	**BIC**	**logLik**	**Dev**	**DF**
Value	2990	3021	-1487	2974	346	2993	3016	-1490	2981	348

Deviance comparisons (WPD3 < WPD4)**:** X^2 = 6.962, DF 2, P = 0.0308

Legend: Inner quadrats = four 10-m by 10-m quadrats centred on each nest. Outer quadrats = 12 10-m by 10-m quadrats surrounding the inner quadrats. Horizontal lines are responses estimated by the model. Sample size = 61 (S1 Dataset).
