# Supplementary material for: Woody plant encroachment drives the decline of a grassland bird: The fate of golden-shouldered parrot (Psephotellus chrysopterygius) nests
Source: PLoS One. 2025 Jul 23;20(7):e0327543. doi: 10.1371/journal.pone.0327543 (PMC12286340; doi:10.1371/journal.pone.0327543)
Supplement: S3 Table — (PDF) [file pone.0327543.s007.pdf]

S3 Table. Negative binomial models explaining the relationship between Bitterlich score and number of stems around golden-shouldered parrot nests.

Coefficient	Estimate	SE	Z	P	VIF	DF	AIC
**Model BITT1:** Full model							
**(Intercept)**	**1.19139**	**0.19880**	**5.99**	**< 0.0001**		**8**	**339.1**
**No. 2-5 cm DBH stems, inner quadrats**	**0.002451**	**0.001169**	**2.10**	**0.0361**	**1.882**		
**No. 5-10 cm DBH stems, inner quadrats**	**0.01797**	**0.00508**	**3.54**	**0.0004**	**2.221**		
No. > 10 cm DBH stems, inner quadrats	-0.002220	0.016791	-0.13	0.8948	1.555		
No. 2-5 cm DBH stems, outer quadrats	-0.0006558	0.0011563	-0.57	0.5706	1.680		
No. 5-10 cm DBH stems, outer quadrats	-0.007090	0.006092	-1.16	0.2446	2.408		
No. > 10 cm DBH stems, outer quadrats	0.01285	0.01665	0.77	0.4404	1.608		
**Model BITT2:** Stepwise model							
**(Intercept)**	**1.184**	**0.158**	**7.491**	**< 0.0001**		**4**	**333.1**
**No. 2-5 cm DBH stems, inner quadrats**	**0.001801**	**0.000878**	**2.051**	**0.0403**	**1.021**		
**No. 5-10 cm DBH stems, inner quadrats**	**0.01415**	**0.00352**	**4.018**	**< 0.0001**	**1.021**		

Legend: Significant terms are highlighted in bold text. VIF = Variance Inflation Factor (threshold for acceptance < 5). Sample size = 61 (S1 Dataset).
