# Supplementary material for: Woody plant encroachment drives the decline of a grassland bird: The fate of golden-shouldered parrot (Psephotellus chrysopterygius) nests
Source: PLoS One. 2025 Jul 23;20(7):e0327543. doi: 10.1371/journal.pone.0327543 (PMC12286340; doi:10.1371/journal.pone.0327543)
Supplement: S4 Table — (PDF) [file pone.0327543.s008.pdf]

S4 Table. Estimated predation rates affecting golden-shouldered parrot nests.

Nest outcome	Cohort lost<br>Eggs (% nests)	Chicks (% nests)	Adults (% nests)	All stages (% nests)
**Likely predation by reptile**				
No eggs hatched	2.8		1.9	3.7
No chicks fledged		3.7	0.9	3.7
**Subtotal**	2.8	3.7	2.8	7.4
**Possible predation by reptile**				
No eggs hatched	13.0		9.3	13.0
No chicks fledged		9.3	5.6	9.3
**Subtotal**	13.0	9.3	14.8	22.2
**Likely predation by butcherbird**				
No eggs hatched			0.9	0.9
No chicks fledged		2.8	2.8	4.6
Some chicks fledged		5.6	7.4	9.3
All clutch fledged			1.9	1.9
**Subtotal**		8.3	13.0	16.7
**Possible predation by unknown predator**				
No eggs hatched	1.9			1.9
No chicks fledged			0.9	0.9
Some chicks fledged	4.6	5.6	0.9	10.2
**Subtotal**	6.5	5.6	1.9	13.0
**Total likely predation**				
No eggs hatched	2.8		3.7	4.6
No chicks fledged		6.5	3.7	7.4
Some chicks fledged	1.9	5.6	7.4	9.3
All clutch fledged			1.9	1.9
**Subtotal**	4.6	12.0	16.7	23.1
**Total possible predation**				
No eggs hatched	13.9		9.3	14.8
No chicks fledged		9.3	6.5	10.2
Some chicks fledged	4.6	5.6	0.9	10.2
**Subtotal**	18.5	14.8	16.7	35.2
Total	23.1	26.9	33.3	58.3

Legend: Likely predation involved direct evidence. Possible predation included disappearance of eggs or chicks and/or abandonment by adults. Unknown predators could include butcherbirds or reptiles. Sample size = 108 (S2 Dataset).
