# Supplementary material for: Woody plant encroachment drives the decline of a grassland bird: The fate of golden-shouldered parrot (Psephotellus chrysopterygius) nests
Source: PLoS One. 2025 Jul 23;20(7):e0327543. doi: 10.1371/journal.pone.0327543 (PMC12286340; doi:10.1371/journal.pone.0327543)
Supplement: S5 Table — (PDF) [file pone.0327543.s009.pdf]

S5 Table. Logistic models examining the influence of vegetation structure and nest outcomes around golden-shouldered parrot nests.

Model	Type	Model	Variable	Estimate	SE	Statistic*	P	Dev	DF	Fit ** Model	Fit ** Bitterlich	Tjur's R^2
**Any Fledging**												
AF0	Null	AF ~ 1	Intercept	0.4911	0.1983	2.48	0.0133	143.4	107	n/a	n/a	< 0.0001
AF1	Simple	AF ~ B	Intercept	1.038	0.354	2.93	0.0034	139.6	106	90	80	0.0353
			Bitterlich	-0.09533	0.05016	-1.90	0.0574					
**AF2**	**Exposure**	**AF ~ √B + offset (log (D))**	**Intercept**	**-1.088**	**0.610**	**-1.78**	**0.0747**	**121.6**	**106**	**100**	**100**	**0.1762**
			**√Bitterlich**	**-0.6731**	**0.2484**	**-2.71**	**0.0067**					
AF3	Exposure	AF ~ √B + s(x, y) + offset (log (D))	Intercept	-1.950	0.409	-4.76	< 0.0001	122.1	105.65	90	90	0.1770
			√Bitterlich	-0.1277	0.0537	-2.38	0.0175					
			s(x, y)			0.52	0.2238					
AF4	Exposure	AF ~ √B + W + offset (log (D))	Intercept	-2.160	1.201	-1.80	0.0721	120.5	105	100	100	0.1852
			√Bitterlich	-0.6521	0.2506	-2.60	0.0092					
			Week	0.07267	0.07093	1.02	0.3056					
AF5	Exposure	AF ~ √B + 1|Y + offset (log (D))	Intercept	-1.088	0.610	-1.78	0.0747	121.6	105	100	100	0.1762
			√Bitterlich	-0.6731	0.2484	-2.71	0.0067					

ANOVA comparisons

Model 1	Model 2	ΔDev	DF	P	Model 1	Model 2	ΔDev	DF	P***
AF0	AF1	3.80	1	0.0512					
AF0	AF2	21.83	1	< 0.0001	AF1	AF2	18.03	0	n/a
AF0	AF3	21.29	1.35	< 0.0001	AF2	AF3	-0.53	0.351	n/a
AF0	AF4	22.92	1	< 0.0001	AF2	AF4	1.09	1	0.2955

Model	Type	Model	Variable	Estimate	SE	Statistic*	P	Dev	DF	Fit ** Model	Fit ** Bitterlich	Tjur's R^2
				**Total Nest Success**								
TNS0	Null	TNS ~ 1	Intercept	-0.7351	0.2056	-3.58	0.0003	136.1	107	n/a	n/a	< 0.0001
TNS1	Simple	TNS ~ B	Intercept	-0.1140	0.3608	-0.32	0.7522	131.6	106	80	90	0.0389
			Bitterlich	-0.1188	0.0603	-1.97	0.0490					
TNS2	Exposure	TNS ~ √B + offset (log (D))	Intercept	-2.394	0.607	-3.94	0.0001	129.4	106	90	~100	0.1039
			√Bitterlich	-0.7415	0.2723	-2.72	0.0065					
TNS3	Exposure	AF ~ √B + s(x, y) + offset (log (D))	Intercept	-3.213	0.390	-8.23	< 0.0001	131.7	106	90	~100	0.0891
			√Bitterlich	-0.1450	0.0650	-2.23	0.0256					
			s(x, y)			0.00	0.6704					
**TNS4**	**Exposure**	**AF ~ √B + W + offset (log (D))**	**Intercept**	**-3.253**	**1.135**	**-2.87**	**0.0042**	**128.6**	**105**	**100**	**~100**	**0.1123**
			**√Bitterlich**	**-0.7456**	**0.2780**	**-2.68**	**0.0073**					
			**Week**	**0.06026**	**0.06747**	**0.89**	**0.3718**					
TNS5	Exposure	AF ~ √B + 1|Y + offset (log (D))	Intercept	-3.253	1.135	-2.87	0.0042	128.6	104	100	~100	0.1123
			√Bitterlich	-0.7456	0.2780	-2.68	0.0073					
			Week	0.06026	0.06747	0.89	0.3718					

ANOVA comparisons

Model 1	Model 2	ΔDev	DF	P	Model 1	Model 2	ΔDev	DF	P***
TNS0	TNS1	4.47	1	0.0346					
TNS0	TNS2	6.66	1	0.0099	TNS1	TNS2	2.19	0	n/a
TNS0	TNS3	4.39	1	0.0362	TNS2	TNS3	-2.27	< 0.0001	n/a
TNS0	TNS4	7.46	1	0.0240	TNS2	TNS4	0.80	1	0.3703

Legend: * Statistic is Z, except for smooth term s(x, y), when it is F. ** Fit = percentage of binned residuals within 95% confidence intervals (S1 Fig, S2 Fig). *** P cannot be computed for comparisons with DF < 1. AF = Any Fledging, B = Bitterlich score, D = days of observation, Dev = Residual deviance, s(x, y) = spatial smooth, TNS = Total Nest Success, W = Week, Y = Year, n/a = not applicable. Best-performing models highlighted in bold. Sample size = 108 (S2 Dataset).
