# Supplementary material for: Woody plant encroachment drives the decline of a grassland bird: The fate of golden-shouldered parrot (Psephotellus chrysopterygius) nests
Source: PLoS One. 2025 Jul 23;20(7):e0327543. doi: 10.1371/journal.pone.0327543 (PMC12286340; doi:10.1371/journal.pone.0327543)
Supplement: S6 Table — (PDF) [file pone.0327543.s010.pdf]

S6 Table. Dunn's pairwise comparisons between nesting zones in relation to foliage cover and fire frequency.

Nesting zone 1 (N)	Nesting zone 2 (N)	Statistic	P	Adjusted P
**1990 Autumn Persistent Green**				
Northern degraded (177)	Northern intact (319)	-3.65	0.0003	0.0003
Northern degraded (177)	Southern intact (74)	-12.41	< 0.0001	< 0.0001
Northern intact (319)	Southern intact (74)	-10.66	< 0.0001	< 0.0001
**2000-2020 fire frequency**				
Northern degraded (177)	Northern intact (319)	-3.19	0.0014	0.0014
Northern degraded (177)	Southern intact (74)	-12.57	< 0.0001	< 0.0001
Northern intact (319)	Southern intact (74)	-11.17	< 0.0001	< 0.0001
**2020 Autumn Persistent Green**				
Northern degraded (177)	Northern intact (319)	13.32	< 0.0001	< 0.0001
Northern degraded (177)	Southern intact (74)	15.92	< 0.0001	< 0.0001
Northern intact (319)	Southern intact (74)	7.40	< 0.0001	< 0.0001

Legend: Based on average values in 100 m buffers around nesting zones. Sample size = 570 (S4 Dataset).
Data sources: Autumn Persistent Green: Department of Environment and Science. Seasonal persistent green - Landsat, JRSRP algorithm Version 3.0, Australia coverage. Terrestrial Ecosystem Research Network. Brisbane: Queensland Government; 2023.
Fire frequency: Charles Darwin University. NAFI: North Australian and Rangelands fire information website. Darwin: Charles Darwin University; 2024.
