# Supplementary material for: Woody plant encroachment drives the decline of a grassland bird: The fate of golden-shouldered parrot (Psephotellus chrysopterygius) nests
Source: PLoS One. 2025 Jul 23;20(7):e0327543. doi: 10.1371/journal.pone.0327543 (PMC12286340; doi:10.1371/journal.pone.0327543)
Supplement: S7 Table — (PDF) [file pone.0327543.s011.pdf]

S7 Table. Generalised additive model explaining the influence of vegetation type and initial foliage cover on subsequent fire frequency.

**Model parameters**	
Model FF1	Fire Frequency (2000-2020) ~ Vegetation Type + s(1990 Autmn Persistent Green)
Family	Gaussian
Link function	Identity link
Method	Restricted Maximum Likelihood
Sample size	570 nests (S4 Dataset)
Adjusted r^2	0.305
Deviance explained	31.4 %

**Parametric coefficients**	**Estimate**	**S.E.**	**t**	**P**
(Intercept)	6.811	0.1734	39.29	< 0.0001
		**Vegetation Type**		
**Eucalypt woodland**	-	-	-	-
**Floodplain forest**	-0.6126	0.4428	-1.383	0.1671
**Tea tree woodland**	1.777	0.3058	5.812	< 0.0001
**Heath**	1.267	0.5052	2.508	0.0124
**Grassland**	-2.839	0.5288	-5.369	< 0.0001

Approximate significance of smooth term (basis type = factor smooth)

**Variable**	**Effective DF**	**Reference DF**	**F**	**P**	**k'**	**EDF**	**k-index**	**P**
1990 Autumn Persistent Green	3.573	4.478	25.90	< 0.0001	9	3.57	1.01	0.60

Pairwise comparisons between Vegetation Types with Benjamini-Hochberg adjustment
(t-ratio above diagonal, p-value below diagonal)

	**Eucalypt woodland**	**Floodplain forest**	**Teatree woodland**	**Heath**	**Grassland**
**Eucalypt woodland**		1.38	-5.81	-2.51	5.37
**Floodplain forest**	0.1857		-4.99	-3.02	3.43
**Teatree woodland**	< 0.0001	< 0.0001		0.95	8.17
**Heath**	0.0156	0.0038	0.3415		5.93
**Grassland**	< 0.0001	0.0011	< 0.0001	< 0.0001	

Predicted fire frequency

**1990 Autumn Persistent Green**	**0**	**15**	**30**
Predicted Fire Frequency (2000-2020)	10.5	6.2	5.2

Data sources: Fire frequency: Charles Darwin University. NAFI: North Australian and Rangelands fire information website. Darwin: Charles Darwin University; 2024.

Vegetation type: Department of Environment and Science. Remnant 2021 Broad Vegetation Groups — Queensland. Version 6 ed. Brisbane: Queensland Government; 2023.

Autumn Persistent Green: Department of Environment and Science. Seasonal persistent green - Landsat, JRSRP algorithm Version 3.0, Australia coverage. Terrestrial Ecosystem Research Network. Brisbane: Queensland Government; 2023.
