# Supplementary material for: Woody plant encroachment drives the decline of a grassland bird: The fate of golden-shouldered parrot (Psephotellus chrysopterygius) nests
Source: PLoS One. 2025 Jul 23;20(7):e0327543. doi: 10.1371/journal.pone.0327543 (PMC12286340; doi:10.1371/journal.pone.0327543)
Supplement: S8 Table — (PDF) [file pone.0327543.s012.pdf]

S8 Table. Generalised additive model explaining influence of vegetation type, initial foliage cover and fire frequency on subsequent change in foliage cover.

**Model parameters**	
Model FCC1	Change in Autmn Persistent Green ~ Vegetation Type + s(1990 Autmn Persistent Green) + s(Fire Frequency (2000-2020))
Family	Gaussian
Link function	Identity link
Method	Restricted Maximum Likelihood
Sample size	570 nests (S4 Dataset)
Adjusted r^2	0.113
Deviance explained	12.7%

**Parametric coefficients**	**Estimate**	**S.E.**	**t**	**P**
(Intercept)	9.894	0.2672	37.03	< 0.0001
		**Vegetation Type**		
**Eucalypt woodland**	-	-	-	-
**Floodplain forest**	-2.292	0.6834	-3.35	0.0009
**Tea tree woodland**	-0.2583	0.4834	-0.53	0.5933
**Heath**	3.030	0.7809	3.88	0.0001
**Grassland**	0.1118	0.8310	0.14	0.8930

Approximate significance of smooth term (basis type = factor smooth)

**Variable**	**Effective DF**	**Reference DF**	**F**	**P**	**k'**	**EDF**	**k-index**	**P**
1990 Autumn Persistent Green	3.687	9	3.346	< 0.0001	9	3.69	1.01	0.64
Fire Frequency (2000-2020)	1.366	9	0.575	0.0203	9	0.95	0.95	0.08

Pairwise comparisons between Vegetation Types with Benjamini-Hochberg adjustment
(t-ratio above diagonal, p-value below diagonal)

	**Eucalypt woodland**	**Floodplain forest**	**Teatree woodland**	**Heath**	**Grassland**
**Eucalypt woodland**		3.35	0.53	-3.88	-0.13
**Floodplain forest**	0.0021		-2.71	-5.53	-2.38
**Teatree woodland**	0.7416	0.0132		-3.99	-0.40
**Heath**	0.0004	< 0.0001	0.0004		2.67
**Grassland**	0.8930	0.0253	0.7625	0.0132	

Predicted change in Autumn Persistent Green (1990-2020)

	**Fire Frequency (2000-2020)**		
**1990 Autumn Persistent Green**	0	5	15
0	8.5	8.5	7.0
17	11.5	11.5	10.1
30	8.5	8.5	7.1

Data sources: Autumn Persistent Green: Department of Environment and Science. Seasonal persistent green - Landsat, JRSRP algorithm Version 3.0, Australia coverage. Terrestrial Ecosystem Research Network. Brisbane: Queensland Government; 2023.

Fire frequency: Charles Darwin University. NAFI: North Australian and Rangelands fire information website. Darwin: Charles Darwin University; 2024.

Vegetation type: Department of Environment and Science. Remnant 2021 Broad Vegetation Groups — Queensland. Version 6 ed. Brisbane: Queensland Government; 2023.
