# Supplementary figures and images for: Woody plant encroachment drives the decline of a grassland bird: The fate of golden-shouldered parrot (Psephotellus chrysopterygius) nests
Source: PLoS One. 2025 Jul 23;20(7):e0327543. doi: 10.1371/journal.pone.0327543 (PMC12286340; doi:10.1371/journal.pone.0327543)

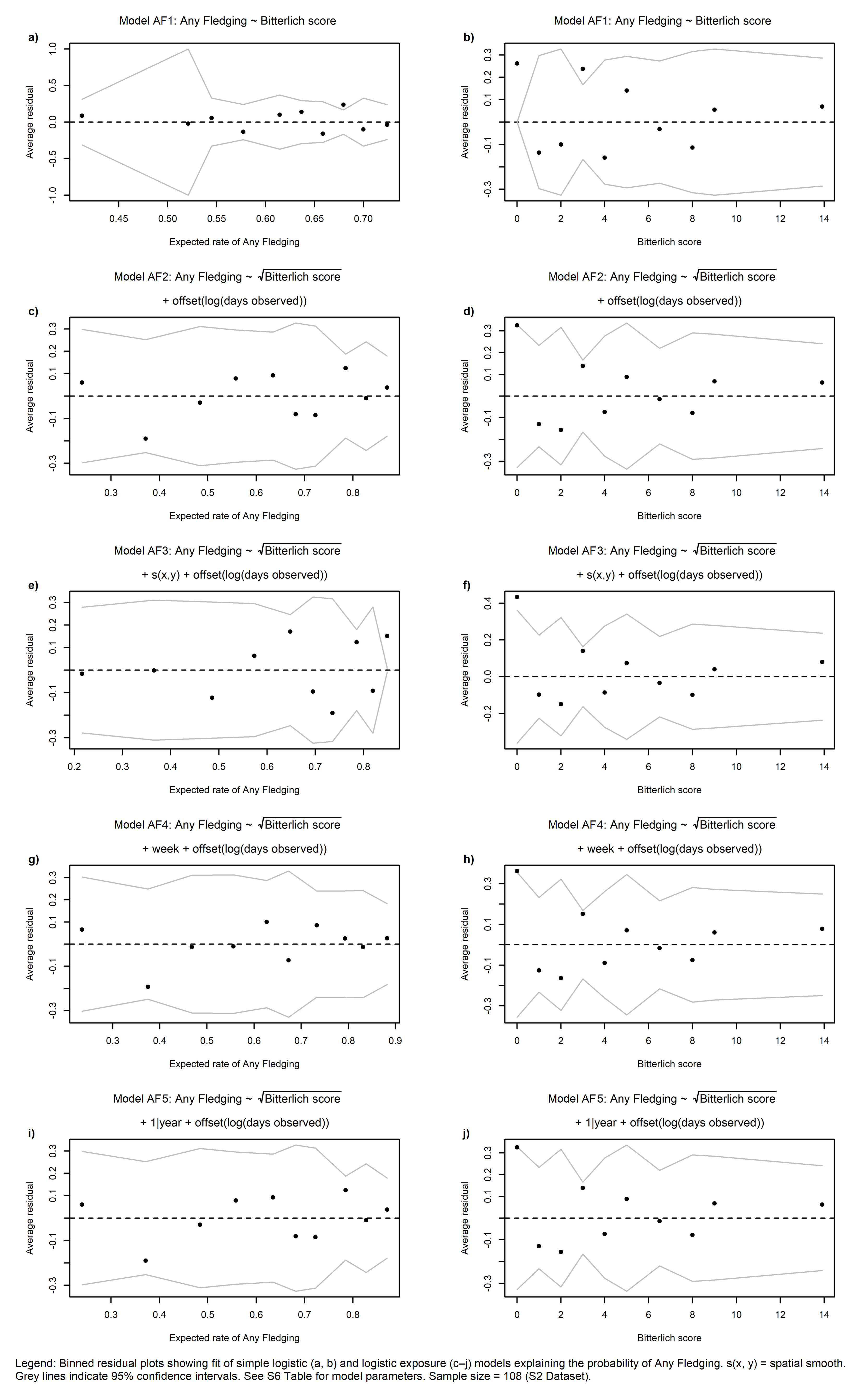

Supplement: S1 Fig — (TIF) [file pone.0327543.s013.tif]

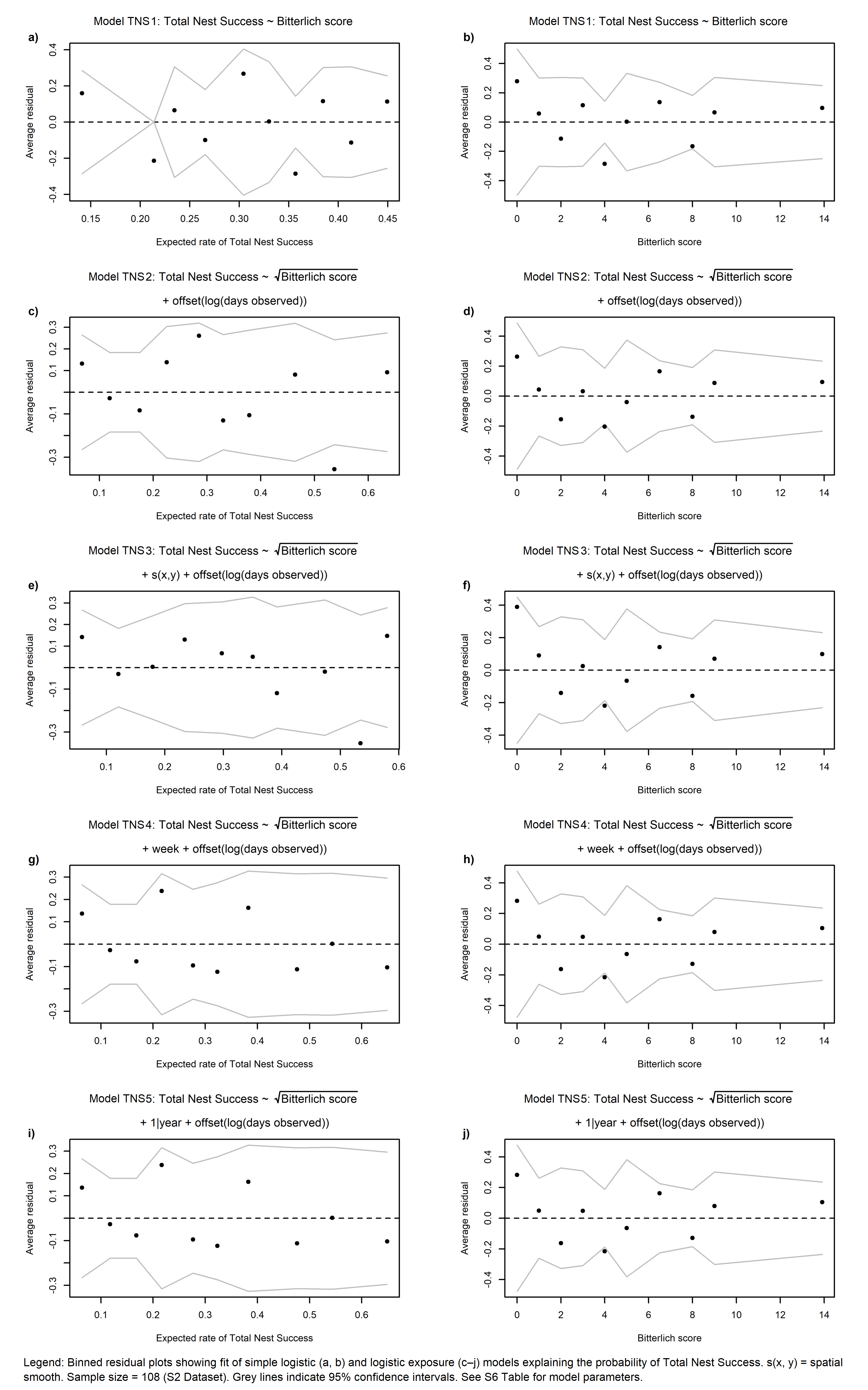

Supplement: S2 Fig — (TIF) [file pone.0327543.s014.tif]

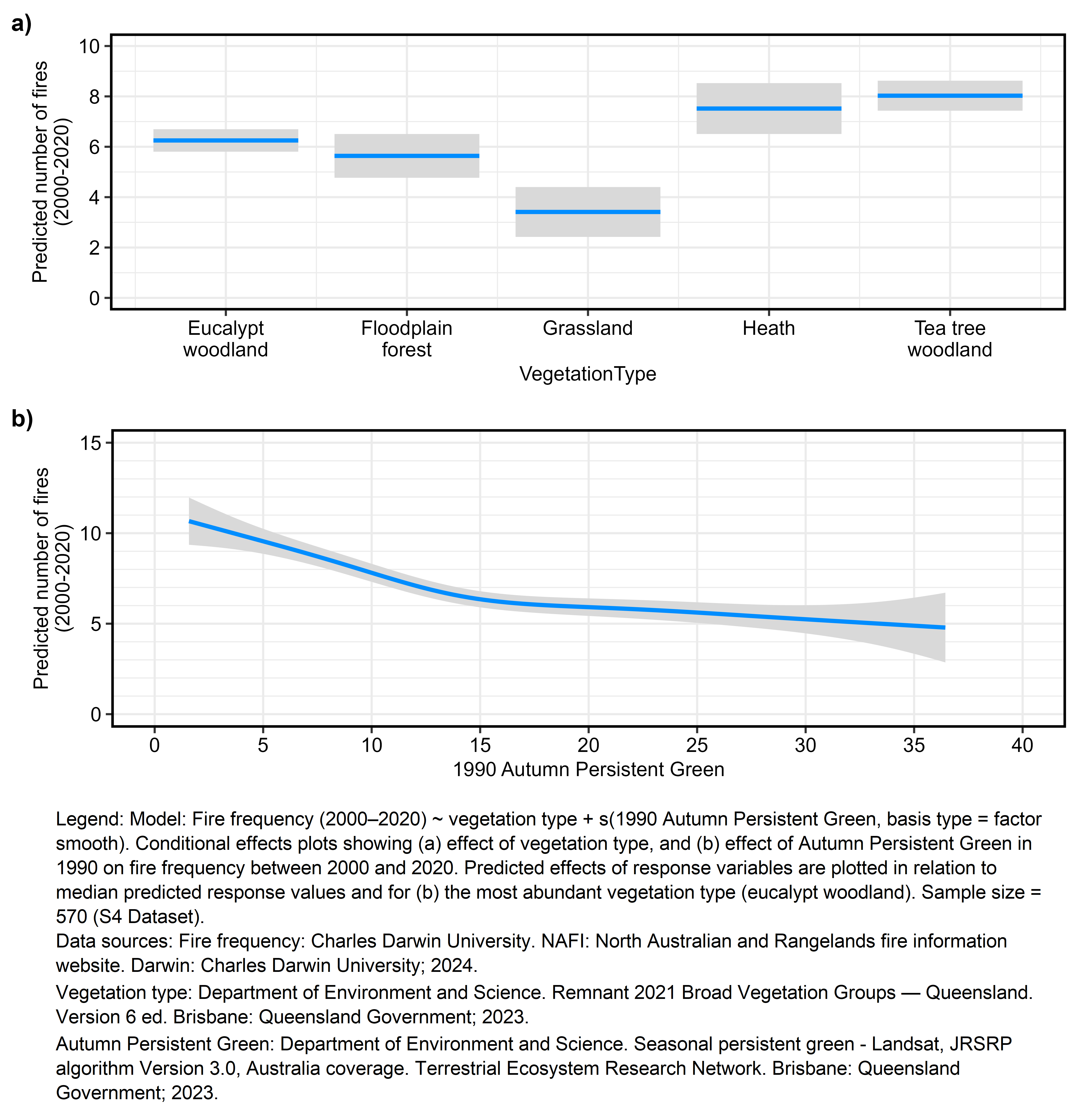

Supplement: S3 Fig — (TIF) [file pone.0327543.s015.tif]

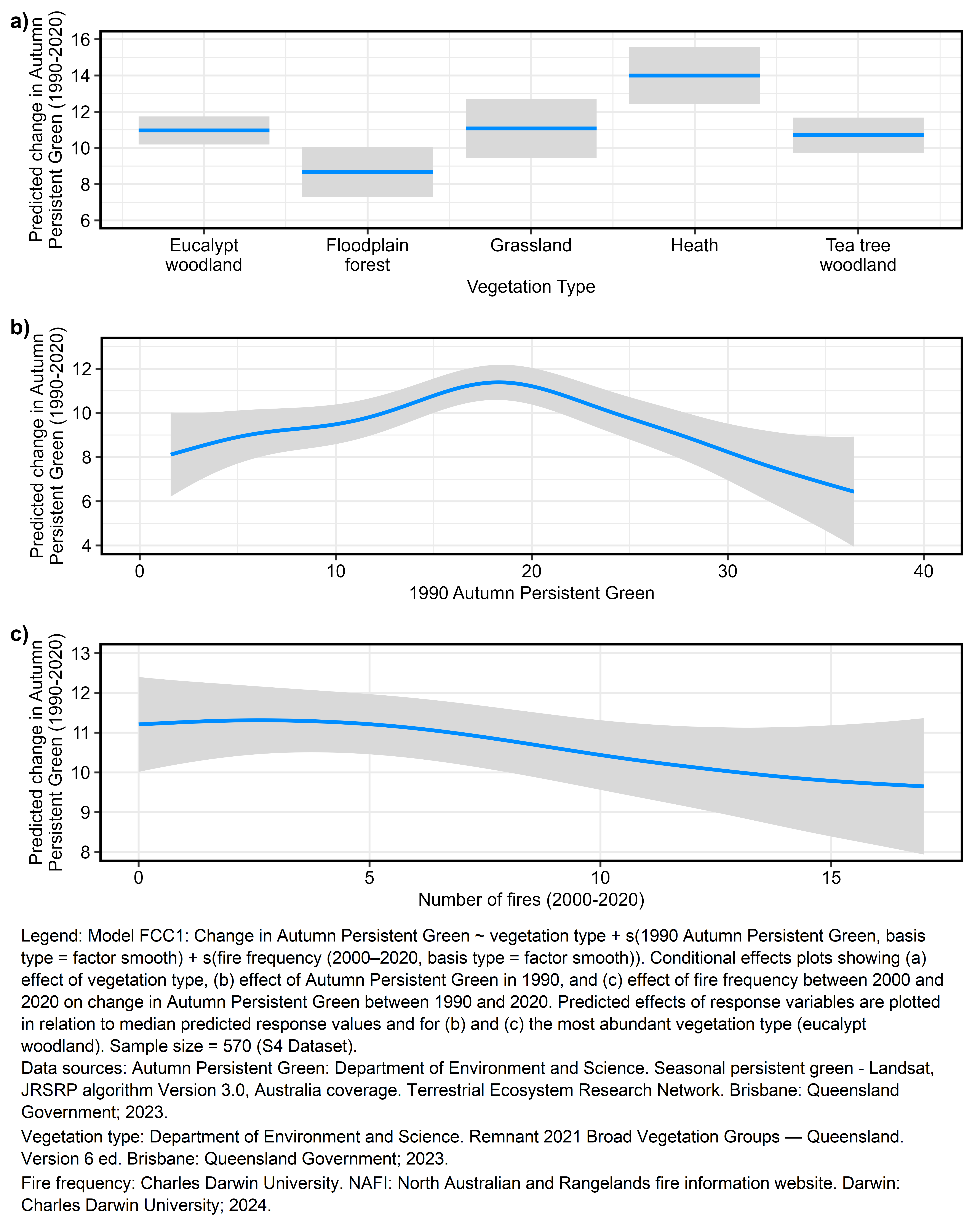

Supplement: S4 Fig — (TIF) [file pone.0327543.s016.tif]
